# 胸部恶性肿瘤围术期静脉血栓栓塞症预防中国专家共识（2018版）

**DOI:** 10.3779/j.issn.1009-3419.2018.10.03

**Published:** 2018-10-20

**Authors:** 辉 李, 格宁 姜, 中国胸外科静脉血栓栓塞症研究协作组

**Affiliations:** 1 100020 北京，首都医科大学附属北京朝阳医院胸外科 Department of Thoracic Surgery, Beijing Chaoyang Hospital, Capital Medical University, Beijing 100020, China; 2 200433 上海，同济大学附属上海市肺科医院 Department of Thoracic Surgery, Shanghai Pulmonary Hospital, Tongji University, Shanghai 200433, China

静脉血栓栓塞症（venous thromboembolism, VTE）是外科术后常见并发症和医院内非预期死亡的重要危险因素，也是恶性肿瘤患者的第二大死亡原因^[1-6]^。

肺癌和食管癌是两类最常见的胸部恶性肿瘤，其中肺癌的发病率和死亡率在我国恶性肿瘤中均占第1位；而食管癌发病率占第6位，死亡率占第4位^[7]^。

研究^[8-13]^已证实肺癌和食管癌均是VTE的高危因素，特别是围术期VTE发生率较高。因此，如何降低胸部恶性肿瘤围术期VTE发生率是我国胸外科医生面临的严峻挑战。

尽管国内外已有众多有关VTE防治的共识和指南^[6, 14-25]^，但针对胸部恶性肿瘤围术期VTE预防的共识尚属空白。鉴于此，中国胸外科静脉血栓栓塞研究协作组成立了一个“胸部恶性肿瘤围术期静脉血栓栓塞症预防中国专家共识”工作组，其目的是规范胸部恶性肿瘤患者VTE预防的流程及方法，以期降低围术期VTE发生率。该工作组由来自不同专业（胸外科、肺部肿瘤外科、呼吸及危重症医学科、血管外科、药理学及护理学）的31名专家组成。

## 1 概述

### 1.1 VTE基本概念

#### 1.1.1 VTE

指血液在静脉内不正常的凝结，从而导致血管完全或不完全阻塞，属静脉回流障碍性疾病。VTE包括两种类型：深静脉血栓形成（deep vein thrombosis, DVT）和肺栓塞（pulmonary embolism, PE），这两种类型是VTE在不同部位和不同阶段的两种临床表现形式^[6, 26, 27]^。

#### 1.1.2 DVT

可发生于全身各部位静脉，尤以下肢深静脉为多。下肢近端（腘静脉或其近侧部位，包括股静脉、髂静脉等）DVT是PE血栓栓子的主要来源。下肢远端的小腿肌间静脉丛由于分支多、管径细、管壁薄、静脉瓣数量较少和血流速度缓慢，以及周围为无深筋膜等坚硬组织等原因，因而也容易形成血栓，因此目前认为肌间静脉血栓也是DVT的一种特殊类型^[28]^。

#### 1.1.3 PE

是指内源性或外源性栓子阻塞肺动脉引起肺循环障碍的一组疾病或临床综合征的总称，包括肺血栓栓塞症（pulmonary thromboembolism, PTE）、脂肪栓塞、羊水栓塞、空气栓塞以及肿瘤栓塞等。由于PE中最常见的是PTE，因此国内外相关指南中多使用PE一词特指PTE。

### 1.2 胸部恶性肿瘤VTE流行病学

大量循证医学证据证明，VTE是一种严重威胁癌症患者生活质量和生存状况的常见疾病，在接受外科手术和化疗的恶性肿瘤患者中尤为突出^[27, 29]^。有报道^[29, 30]^证实，首次住院的癌症患者中VTE的发病率按照肿瘤部位的不同约为3%-12%。在一项关于外科癌症患者血栓预防的临床试验中，未经抗肿瘤治疗的患者其DVT的发生率平均为29%^[29]^。在另一项纳入17, 284例实体肿瘤患者的研究中，门诊化疗后12个月内VTE的发生率为12.6%^[31]^。

早在20多年前就有关于肺癌与VTE之间相关性的报道^[10, 32]^。一项针对肺癌患者的大型流行病学研究纳入了91, 933例新诊断肺癌患者，研究发现肺癌诊断后1年和2年内累计VTE发生率分别为3.0%和3.4%^[33]^。另一项回顾性研究对比了6, 732例肺癌患者和17, 284例非肺癌组，发现肺癌组VTE的发生率为13.9%，而非肺癌组仅为1.4%^[34]^。我国一项流行病学调查证实，在新诊断肺癌患者中VTE发生率为13.2%^[35]^。

随后国内外的临床研究进一步证实了肺癌手术与VTE发生的相关性，研究结果发现肺癌术后VTE的发生率约为7.3%-13.9%^[26, 36]^。Ziomek等^[37]^报道了肺癌切除手术患者VTE发生率为26%（15例DVT，5例PE）。Agzarian等^[38]^的研究发现，肺癌手术患者VTE的发病率为12.1%，而其中大部分患者为无症状VTE事件，并且均发生在出院以后。Christensen等^[39]^在一项荟萃分析中回顾了近60年来已发表的19项相关研究，结果发现肺癌术后VTE的总发生率为2.0%（0.2%-19%）。Mason等^[40]^报道了全肺切除术后VTE发生率为7.4%。2015年我国报道了一项对94例非小细胞肺癌患者全肺切除标本的回顾性研究，结果发现约59.6%的患者存在血栓栓塞性事件，包括25%的肺动脉血栓形成，33%的肺静脉血栓形成，而发现血栓形成的标本中，53.6%合并脉管瘤栓或血管受侵^[41]^。在新近发表的一项中国单中心前瞻性队列研究中，未经VTE预防的胸外科术后患者，VTE总发生率为13.9%，而肺癌术后VTE发生率高达16.4%^[42]^。

目前关于食管癌发生VTE的流行病学资料不多，而且仅限于有症状的患者，因此该人群术后VTE的真实发病率可能被低估。仅有的几项研究^[43, 44]^发现，食管癌切除术围手术期症状性VTE发生率为5%-14%。研究^[29, 45]^还发现，一旦发生VTE，食管癌术后住院患者死亡率从6.9%增加到13.6%。

2015年美国外科医师学会主持了一项针对五种恶性肿瘤手术患者术后并发症的大型回顾性研究，共有74, 361例患者接受了评估，其中包括6, 849例肺癌患者和3, 126例食管癌患者。研究^[46]^发现，肺癌术后VTE发生率为1.8%，食管癌术后则为5.9%。食管癌术后VTE发生率在所统计的五种癌症中最高。

**推荐意见1：肺癌和食管癌患者是VTE高危人群，术后VTE的发生率较高。**

### 1.3 恶性肿瘤患者易患VTE的病理生理基础

德国病理学家Rudolf Virchow在1856年提出血栓形成三要素：血管内皮损伤、血流淤滞和高凝状态^[47]^。

恶性肿瘤本身就是VTE高危因素之一，特别是接受手术和放化疗的患者。恶性肿瘤可以通过很多机制破坏血管系统纤维蛋白沉积与降解之间的平衡。恶性肿瘤患者多有凝血机制的异常，表现为纤维蛋白降解产物增高，血小板增多，高纤维蛋白原血症，凝血酶原时间延长，提示有慢性弥漫性血管内凝血的存在。此外，肿瘤组织本身亦能分泌促凝物质，如组织因子、促血小板聚集物质、多糖蛋白及血浆纤维蛋白溶酶原激活剂等，使机体处于高凝状态，具有高血栓形成的倾向。而被癌细胞浸润的血管内膜因丧失了抗血栓形成的能力，较易在血管壁和心瓣膜上形成血栓和赘生物^[48, 49]^，这些均是恶性肿瘤患者VTE高发的病理生理基础。

### 1.4 中国胸外科VTE防治现状

尽管国内外的VTE预防指南均推荐对高危恶性肿瘤患者采取预防性治疗措施^[5, 6, 15, 17, 18, 21, 25, 50]^，但在我国，胸部恶性肿瘤患者接受规范的VTE预防的比例还较低，其主要的原因是：①大多数中国胸外科医生对VTE认知度和关注度不够；②依据以往的国内散在报道，认为VTE发生率较低；③担心药物预防后大出血的风险^[51]^。

## 2 静脉血栓栓塞症的危险因素及评价方法

### 2.1 危险因素

胸部恶性肿瘤手术患者的VTE危险因素包括围术期和手术相关两大部分。围术期危险因素包括：既往VTE史、手术、创伤、卧床、恶性肿瘤及肿瘤治疗史、高龄、内科基础疾病（特别是呼吸系统合并症）、肥胖、吸烟、下肢静脉曲张、严重感染、使用镇静剂、遗传性或获得性血栓形成倾向等。手术相关危险因素包括：中心静脉置管和其他侵入措施的应用、麻醉药物的应用、手术时间、手术方式以及机械通气等。这些围术期及手术相关危险因素常同时存在（表 1）^[52]^。

**1:** VTE独立危险因素^[53]^ Independent risk factors of VTE^[53]^

Characteristic	Odds ratio	95%CI	*P*
Patient age	1.24	0.88-1.73	0.215, 7
Body mass index (kg/m^2^)	1.08	1.05-1.11	< 0.000, 1
Major surgery	18.95	9.22-38.97	< 0.000, 1
Hospitalization for acute medical illness	5.07	3.12-8.23	< 0.000, 1
Trauma/fracture	4.56	2.46-8.46	< 0.000, 1
Active cancer	14.64	7.73-27.73	< 0.000, 1
Neurologic Disease with leg paresis	6.10	1.97-18.89	0.001, 7
Varicose veins	1.22	0.89-1.68	0.223, 9
VTE: venous thromboembolism.			

胸部恶性肿瘤，特别是接受外科手术的肺癌和食管癌患者，已被证实是发生VTE的高危人群。有研究认为，VTE的发生与肺癌患者的个体因素密切相关，包括病理类型、肿瘤分期以及手术方式等相关（例如楔形切除、肺叶切除、肺切除、袖状切除等）^[54]^。一项研究发现，VTE的发生率在原发性支气管肺癌（25%）明显高于转移性肿瘤和良性病变（0%）；腺癌（44%）明显高于其他病理类型肿瘤（12%）；直径超过3 cm的原发性肺癌（47%）明显高于小肺癌（15%）；Ⅱ期肺癌（50%）明显高于I期肺癌（21%）；全肺切除和肺叶切除患者（29%）明显高于段或楔形切除患者（4%）^[37]^。

### 2.2 危险因素评估

虽然已有循证医学证据证实恶性肿瘤患者发生VTE的风险很高，但这些人群中的亚组之间的风险差异很大。因此，区分低危和高危患者对于优化血栓预防的风险-受益比率至关重要。

现有的VTE风险评估模型有多种，包括Caprini风险评估量表（Caprini risk assessment model, Caprini RAM）^[55]^、Rogers评分量表^[56]^、Padua评分量表^[57]^和Khorana评分量表^[58]^。所有这些都是专门为评估VTE的风险而设计的评估工具。美国胸科医师学会（American College of Chest Physicians, ACCP）非骨科外科患者VTE预防临床实践指南第9版（ACCP-9）推荐在外科患者中使用Caprini和Rogers评估量表^[18]^，而Padua评分被认为是最适合内科住院患者的风险评估模型^[59]^，Khorana评分是一种预测化疗患者VTE风险的模型^[58]^。

外科临床最常用的Caprini RAM是个体化的VTE风险评估量表，目前应用范围很广，包括普通外科、骨科、妇产科以及肿瘤科等。经典的Caprini RAM包括大约40个危险因素（包括先天性和/或后天性危险因素），几乎涵盖了住院患者VTE的所有危险因素，每个危险因素根据危险程度的不同被赋予1分-5分不同的分值，最后根据得到的累计分数将患者VTE发生风险分为极低危（0分）、低危（1分-2分）、中危（3分-4分）和高危（≥5分）4个等级，不同的风险等级推荐不同的VTE预防措施^[55, 60-62]^。因此，该量表不仅可以预测VTE的风险，而且还推荐相应的预防措施，包括特定预防措施的类型和持续时间。但在临床实践中，这种经典Caprini风险评估标准并不适合胸部恶性肿瘤患者，因为按此标准，几乎所有住院的胸部恶性肿瘤患者都达到了高危风险。因此近年来，一种改良的Caprini RAM已经被国外胸外科医师所使用。改良的Caprini量表将风险分级简化为3个级别：低危（0分-4分）、中危（5分-8分）和高危（≥9分）（表 2），被认为更适用于胸部恶性肿瘤患者^[63-65]^。

**2:** 改良Caprini量表（VTE风险评分） Modified Caprini risk assessement model

Caprini risk factor	Caprini score
Age 40-59 (yr)	1
Abnormal pulmonary function	1
Acute myocardial infarction (< 1 mo)	1
Body mass index ≥30 (kg/m^2^)	1
Congestive heart failure (< 1 mo)	1
History of inflammatory bowel disease	1
History of prior major surgery (< 1 mo)	1
Complications of pregnancy	1
Oral contraceptive use or HRT	1
Sepsis (< 1 mo)	1
Serious acute lung disease (< 1 mo)	1
Swollen legs (current)	1
Varicose veins	1
Age 60-74 (yr)	2
Central venous access	2
Confined to bed (> 72 h)	2
Major open surgery (≥45 min)	2
Present cancer	2
Prior cancer, except nonmelanoma skin	2
Age ≥75 (yr)	3
History of VTE	3
Family history of VTE	3
Chemotherapy	3
Positive anticardiolipin antibody	3
Positive Lupus anticoagulant	3
cAcute spinal cord injury (< 1 mo)	5
Major surgery ≥6 h	5

**推荐意见2：对因胸部恶性肿瘤住院的患者应进行危险因素分层，锁定高危人群。推荐使用改良Caprini风险评估表进行动态评估。**

## 3 VTE的临床表现

### 3.1 急性DVT的临床表现

#### 3.1.1 患肢肿胀

是DVT最常见的症状，患肢组织张力高，呈非凹陷性水肿。皮肤泛红，皮温较健侧高。肿胀严重时，皮肤可出现水疱。随血栓部位的不同，肿胀部位也有差异。髂-股静脉血栓患者，整个患侧肢体肿胀明显；而小腿静脉丛血栓患者，肿胀仅局限在小腿。

#### 3.1.2 疼痛和压痛

血栓在静脉内引起炎症反应和血栓堵塞静脉引起的下肢静脉回流受阻，可使患肢局部产生持续性疼痛，直立时疼痛加重。压痛主要局限在静脉血栓产生炎症反应的部位。

#### 3.1.3 浅静脉曲张

浅静脉曲张属于代偿性反应。

#### 3.1.4 PE

血栓脱落可引起PE。急性PE可严重威胁生命，需紧急就医抢救。

### 3.2 急性PE的临床表现

#### 3.2.1

急性PE的临床分型：急性PE的临床分型主要依据患者的血流动力学状况、心肌损伤标记物水平和右心室功能三个方面进行综合评估确定。急性PE患者合并血流动力学不稳定为高危，血流动力学稳定但合并右心室功能不全和（或）心肌损伤标记物升高为中危，而血流动力学稳定且无右心室功能不全和心肌损伤标记物升高为低危^[66]^。

#### 3.2.2

急性PE临床表现：PE的临床表现常因肺动脉血栓栓塞的部位不同而明显不同：①小面积PE（栓塞面积小于20%）可无明显症状，或仅有发热、短暂气急、胸背疼痛、咳嗽、咯血、心悸、多汗或血压下降等不典型症状；②大块或多发性PE（栓塞面积大于50%），可出现典型的呼吸困难、胸痛、咯血和/或循环衰竭三联症；③猝死型肺栓塞：术后短期内发生大面积PE，常发生猝死。其在胸外科手术患者中多表现为术后7天内下床活动后突发的呼吸困难、晕厥甚至心跳骤停。

对于胸外科手术患者，任何微小的可疑栓塞表现都应该密切监测和评估。如患者术后出现以下几种临床表现应高度警惕PE的存在：①自主呼吸时，低氧血症进一步恶化；②具有慢性肺部病变和已知的二氧化碳潴留的患者，出现呼吸困难和低氧血症加重，动脉血二氧化碳分压下降；③不明原因的发热；④在血流动力学监测期间，肺动脉压和中心静脉压突然升高。

## 4 VTE诊断方法

### 4.1 VTE评估量表

在临床也常用到一些量表来帮助我们诊断VTE，例如对疑似DVT的患者，可采用Wells-DVT评分量表（表 3）；对于疑似急性PE的患者，常使用简化Wells-PE评分量表（表 4）^[67, 68]^。

**3:** Wells-DVT评估量表 Revised Wells score creteria for assessment of suspected DVT

Creteria	Score (point)
Active cancer (treatment ongoing or within the last 6 months or palliative)	1
Calf swelling > 3 cm compared to the other calf (measured 10 cm below the tibial tuberosity)	1
Collateral superficial veins (non-varicose)	1
Pitting edemas (greaterin the symptomatic leg)	1
Swelling of the entire leg	1
Localized tenderness along the distribution of the deep venous system	1
Paralysis, paresis, or recent plaster cast immobilization of the lower extremities	1
Recenly bedridden > 3 d, or major sugery in the previous 4 wk	1
Previously documented deep vein thrombosis	1
Alternative diagnosis to DVT as likely or more likely	-2
Interpretation: for dichotomized evaluation (like *vs* Unlike) Score of 2 or higher: DVT is “likely”; Score of less than 2: DVT is “unlikely”. DVT: deep vein thrombosis.	

**4:** 简化Wells-PE评分量表^[69]^ Modified Wells-PE score^[69]^

Clinical feature	Score (point)
Previous DVT/PE	1
Heart rate > 100 beats per minute	1
Immobilisation for more than 3 d or surgery in the previous 4 wk	1
Haemoptysis	1
Active cancer	1
Clinical signs and symptoms of DVT	1
Alternative diagnosis is less likely than PE	-2
Interpretation: for dichotomized evaluation (like *vs* Unlike) Score of 2 or higher: PE is “likely”; Score of less than 2: PE is “unlikely”.	

**推荐意见3：建议临床采用VTE诊断评估量表对疑诊患者进行初步筛查，而后根据评分结果选择相应的检查手段进一步明确诊断。**

### 4.2 DVT

#### 4.2.1 血浆D-二聚体

D-二聚体诊断急性DVT的灵敏度较高（> 99%）， > 500 μg/L（ELISA法）有重要参考价值。D-二聚体检测用于诊断DVT的阳性预测值为31%，但阴性预测值则高达98.6%，更具有临床意义。因此，D-二聚体检测可以作为DVT诊断的排除标准^[70, 71]^。

#### 4.2.2 超声检查

多普勒超声血流检查为无创性检查方法，有助于明确患肢血液回流和供血状况。灵敏度、准确性均较高，是DVT诊断的首选方法，适用于对患者的筛查和监测。在超声检查前，按照Wells-DVT评估量表，可将患有DVT的临床可能性分为非常可能（≥2分）和可能性很小（< 2分）。如连续两次超声检查均为阴性，对于可能性很小的患者可以排除诊断，对于非常可能的患者，建议进一步行螺旋CT静脉成像以明确诊断。

#### 4.2.3 螺旋CT静脉成像

准确性较高，可同时检查腹部、盆腔和下肢深静脉情况。

#### 4.2.4 MRI静脉成像

能准确显示髂、股、腘静脉血栓，但不能较准确地显示小腿静脉血栓。优点是无需使用造影剂。

#### 4.2.5 静脉造影

准确性高，不仅可以有效判断有无血栓、血栓部位、范围、形成时间和侧支循环情况，而且常被用来鉴定其他方法的诊断价值。此法虽最为可靠，但当病情太重时，不必强求完成此项检查，并须考虑到造影本身亦有可能加重病变。

### 4.3 急性PE

#### 4.3.1 血浆D-二聚体

可作为非手术患者急性PE的排除标准^[72]^。然而，由于凝血纤溶系统是由手术本身和基础病理改变以及预先存在的恶性肿瘤激活的，术后患者D-二聚体不同程度升高对于手术患者其临床诊断价值有限。因此，在临床实践中，D-二聚体检测不推荐用于癌症患者或手术患者急性PE的诊断。然而，D-二聚体阴性被认为对排除急性PE有很高的价值。通常D-二聚体低于500 μg/L者可基本排除急性PE。经过年龄校正后的D-二聚体阈值（阈值：50岁以上者年龄×10 μg/L）在老年患者中应用更准确。

#### 4.3.2 螺旋CT肺动脉造影（computed tomography pulmonary angiography, CTPA）

近年来，CTPA越来越多地被应用于临床，并被认为是诊断PE的金标准^[25]^。对PE的诊断特异性 > 95%，阴性预测值很高，建议症状出现后24 h内完成。

#### 4.3.3 核素通气/灌注显像

曾一度被作为首选的检查措施。但因其诊断特异性和敏感性相对于CTPA低，目前临床不常用，仅对妊娠、对比剂过敏或肾衰竭患者，可作为CTPA的替代检查。

#### 4.3.4 肺血管造影

虽然肺动脉造影被认为是诊断PE的金标准，但属于侵入性检查，目前已被CTPA等无创性检查方法所替代。然而，对于临床高度疑诊的巨大PE，且溶栓药物或抗凝药物禁忌的患者，建议直接进行肺动脉造影，并应做好干预准备，这将大大减少检查所需的时间和费用。

#### 4.3.5 超声心动图

确诊急性PE的价值有限，但可以通过评估有无右心室扩大、右心室游离壁运动降低、流出道梗阻等征象提示PE诊断和排除其他心血管疾病。通常用于不宜搬动、生命体征不平稳的高危患者，有助于急性PE的危险分层和预后判断。

#### 4.3.6 心肌损伤标记物

包括血浆肌钙蛋白（cTNI, cTNT）、脑钠肽（brain natriuretic peptide, BNP）和N-末端脑钠肽前体（N-terminal pro brain natriuretic peptide, NT-proBNP），是评价心肌是否损伤的标记物，其水平升高提示心肌损伤严重。临床有一定参考价值。

**推荐意见4：D-二聚体检测可以作为DVT和PE诊断的排除标准。**

**推荐意见5：DVT诊断首选多普勒超声血流检查。对高危患者手术前后均应进行下肢静脉多普勒超声检查。**

**推荐意见6：PE诊断首选CTPA。**

## 5 胸部恶性肿瘤围术期VTE预防方法

全球而言，目前已经有了多个国际性和地区性的VTE防治指南，其中主要包括美国全国综合癌症网络（National Comprehensive Cancer Network, NCNN）指南^[6]^、ACCP-9 VTE预防指南^[18]^、ACCP-10 VTE治疗指南^[89]^、美国临床肿瘤学会（American Society of Clinical Oncology, ASCO）指南^[15]^、欧洲医学肿瘤学学会（European Society for Medical Oncology, ESMO）指南^[19]^、国际血栓栓塞和癌症倡议（International thromboembolism and Cancer Initiative, ITAC-CME）^[5]^等。所有因癌症住院的患者均被定义为VTE的中-高危风险人群，并且应考虑在围术期进行VTE的预防。

一般而言，预防措施包括基本预防、机械预防和药物预防^[64, 72, 73]^。积极恰当的预防可改善手术患者的预后和降低死亡率^[6, 18, 19, 26, 74]^。

对于胸部恶性肿瘤患者，尤其是VTE高危风险者，积极的围手术期预防措施对于降低VTE的发生率非常重要。最简单的方法是应用改良Caprini评估量表进行风险分层。评估强调动态性，至少在患者入院和术后即刻都要进行单独评估（图 2）。其他特殊情况，如病情发生重大变化或治疗方案改变等，还应进行再评估。

**1:**
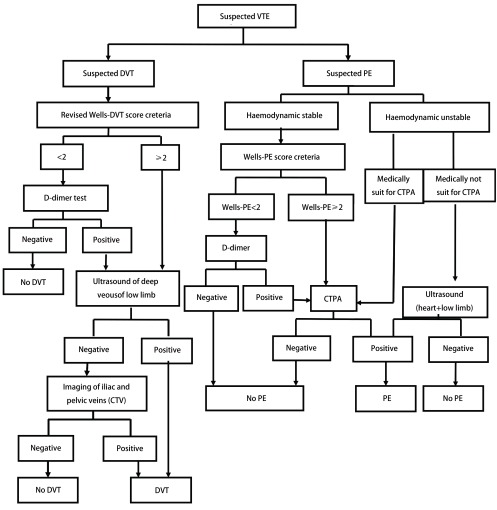
疑诊DVT和PE诊断流程图 Diagram of diagnostic pathway for suspected VTE. CTPA: computed tomography pulmonary angiography; PE: pulmonary embolism.

**2:**
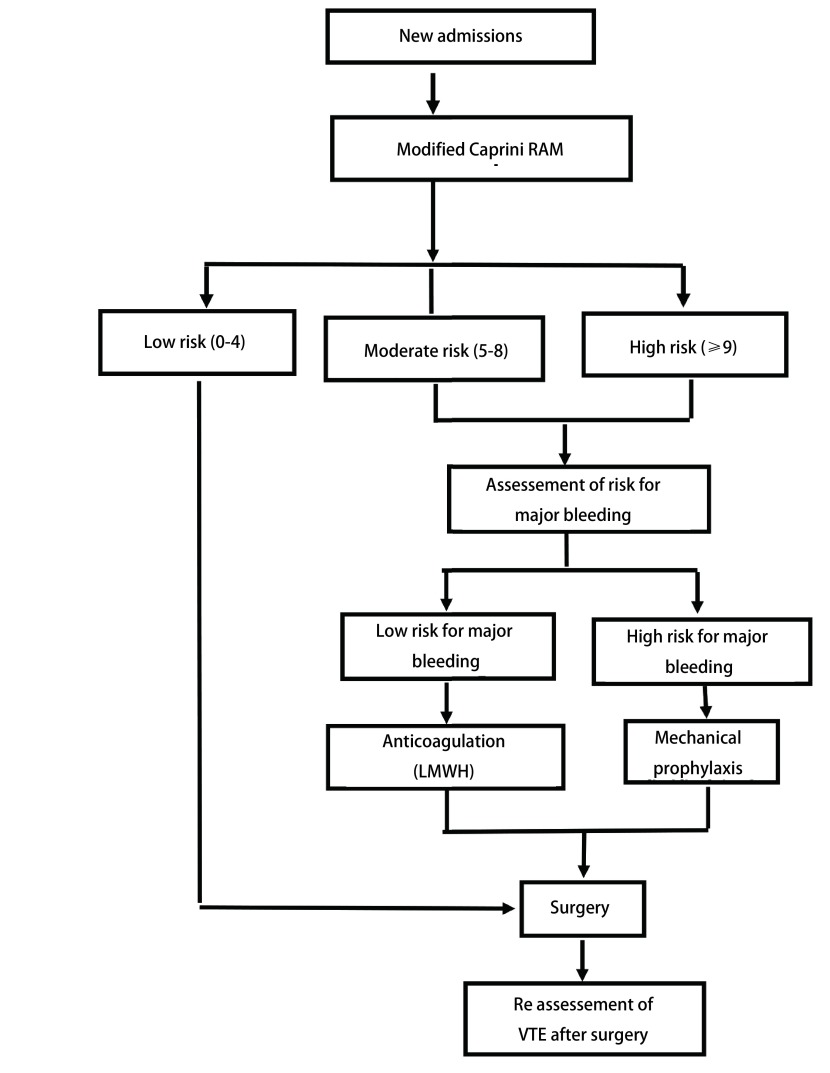
术前VTE风险评估流程图 Algonthm of pre-operative VTE assessement

对于VTE低危患者（0分-4分），建议机械性预防。VTE中危（5分-8分）且大出血低危患者，建议使用低分子肝素（LMWH）7 d-10 d，同时使用机械性预防。VTE高危（≥9分）且大出血低危患者，建议使用LMWH 30 d加机械性预防。VTE中危和高危（≥5分）且大出血高危患者，首先采用机械性预防，一旦大出血风险降低或消失，应立即加用药物预防（表 5）^[5, 6, 18, 21, 25, 64, 74]^。

**5:** 胸外科术后患者VTE预防的推荐意见 Recommendations for thromboprophylaxis in various groups

VTE risk category	Low- or moderate- risk for major bleeding	High risk for major bleeding
Low risk (0-4)	Early ambulation or mechanical prophylaxis	
Moderate risk (5-8)	LMWH (7 d-10 d)+Mechanical prophylaxis	Mechanical prophylaxis
High risk (≥9)	LMWH (30 d)+ Mechanical prophylaxis	Mechanical prophylaxis (IPC), Initiate chemoprophylaxis as soon as the riske of major bleeding diminished

当患者为中-高危（≥5分），但LMWH过敏、患肝素诱导的血小板减少症（heparin induced thrombocytopenia, HIT）或LMWH无法获取时，推荐使用磺达肝癸钠，同时使用机械性预防措施。

### 5.1 基本预防

#### 5.1.1 患者教育

肺癌患者应接受VTE的风险教育。建议患者改善生活方式：戒烟和戒酒，控制血糖或血脂等。

#### 5.1.2 尽早下地活动

鼓励患者尽早下地活动。一些研究已经证实早期下地活动可以防止血栓形成，并降低血栓形成的风险^[62]^。

#### 5.1.3 腿部运动

是最简单、安全和有效的增加静脉血回流到心脏的方法，患者主动的腿部运动或腿部抬高有助于预防下肢深静脉血栓形成^[75]^。对于病情严重、活动困难或需要长期卧床休息的患者，建议使用机械性辅助装置进行腿部被动运动。

#### 5.1.4 避免脱水

围手术期VTE与脱水状态密切相关。因此，除非临床上治疗需要，应避免围术期过度限液^[76]^。

### 5.2 机械预防

机械性预防方法可增加静脉血流和/或减少下肢静脉淤血，包括：梯度压力弹力袜、间歇充气加压装置（intermittent pneumatic compression, IPC）和下肢静脉泵。对于大出血风险和血栓形成风险都很高的患者，建议使用IPC进行机械预防^[6]^。对于VTE高危而出血低-中危的患者，推荐药物预防辅助机械预防。这些设备应尽可能在双腿应用，一旦患者可以下地活动即可停止^[25, 64]^。机械预防不建议作为单一预防措施。因为到目前为止，还没有一种机械预防被证实能降低肿瘤患者发生VTE发生的风险及由PE导致的死亡。

### 5.3 药物预防

#### 5.3.1 药物预防基本原则

ACCP-9指南建议，所有接受扩大肺切除、全肺切除、胸膜外全肺切除或食管切除术的患者均为VTE高危患者，应接受围术期VTE预防，主要方法是术前和术后使用LDUH或LMWH进行血栓预防^[18]^。对于高危患者，还应增加机械性预防^[25]^。然而，药物预防可因其出血的风险导致严重后果，因此，应在权衡出血风险与血栓风险后开始药物预防^[19]^。

#### 5.3.2 药物预防的时机和持续时间

VTE高危的胸部恶性肿瘤患者的抗凝预防应在术前开始（手术前12 h）^[77, 78]^，术后12 h继续给予抗凝预防。术后抗凝预防应维持7 d-10 d^[9, 26, 33, 54, 79]^。对于极高危患者（包括术后残留肿瘤、肥胖或有VTE病史的患者），抗凝预防应延长至术后30 d^[80-88]^。

#### 5.3.3 推荐预防药物

我国现有的抗凝药物包括普通肝素（unfractionated heparin, UFH）、LMWH、磺达肝癸钠、维生素K拮抗剂（vitamin K antagonist, VKA）和直接口服抗凝药物（direct oral anticoagulant drug, NOACs）。LMWH一般被认为是癌症患者首选的抗凝剂，对于不能使用LMWH或UFH的患者，可考虑使用磺达肝癸钠（表 6）^[18, 25, 89]^。

**6:** 预防性抗凝药物的使用方法及疗程 Dosage, route of administration and duration

Anticoaguant		Recommended dose for patients at high risk of VTE
UFH		5, 000 U/s, 10 h-12 h preoperatively, repeated once daily thereafter
LMWH	Nadroparin	2, 850 IU (0.3mL)/s, 12 h preoperatively, repeated 12 h postoperatively, 0.3 mL daily postoperatively for 7 d-10 d.
	Dalteparin	5, 000 IU/s, 12 h preoperatively, then once daily postoperatively for 7 d-10 d.
	Enoxaparin	4, 000 IU (0.4 mL)/s, 12 h preoperatively, 4, 000 U, daily postoperatively for 7 d-10 d.
Indirect Xa factor inhibitor	Fondaparinux	2.5 mg/s, 6 h-8 h postoperatively, once daily extended to 7 d-10 d.
^*^Unfractionated heparin is usually the first choice for preventive drugs in the following cases: ①in patients with severe renal insufficiency; ②in obese patients or underweight patients. #Low molecular weight heparin prophylaxis is 1 time/d, subcutaneous injection.		

##### 5.3.3.1 肝素

目前的数据已经证实，UFH与下肢DVT形成的几率降低和PE的几率降低有关，仅与非致命性大出血的几率增加有关。为了最大限度地减少出血并发症的风险，临床上推荐每天3次使用5, 000 U UFH，剂量需要根据常规活化部分凝血酶原时间（partial prothrombin time, APPT）进行调整。

低分子肝素（low molecular weight heparin, LMWH）：LMWH具有以下特点：①允许根据体重调整剂量，皮下注射，使用方便；②严重出血并发症的发生率相对较低；③不需要常规血液学监测。建议每天使用一次LMWH，以预防肺癌患者术后VTE的发生。LMWH药物预防在术前12 h开始，并至少持续7 d-10 d。不同的LMWH的预防剂量不同，需参考具体药物说明书给药（表 6）。

那屈肝素钙（Nadroparin）推荐剂量为：当患者有中度-高度血栓形成风险（尤其是肿瘤）时，于术前12 h给药0.3 mL，相当于2, 850 IU，其后每日1次皮下注射0.3 mL。

达肝素钠（Dalteparin）起始剂量术前12 h 5, 000 U，术后12 h-24 h（除外出血倾向后皮下给予常规剂量；或术后4 h-6 h给予常规剂量的一半，次日恢复至常规剂量。

依诺肝素钠（Enoxaparin）推荐剂量为：术前12 h给药0.4 mL，相当于40 mg依诺肝素钠，其后每日一次皮下注射0.4 mL。

##### 5.3.3.2 磺达肝癸钠

磺达肝癸钠是一种间接Xa因子抑制剂，其特点是治疗窗口宽，剂量固定。在磺达肝癸钠用药期间不需要常规的血液监测，亦可用于肝素诱导的血小板减少症。与LMWH相比，磺达肝癸钠可显著降低静脉血栓形成的发生率，而不增加大出血的风险。然而，目前还没有应用磺达肝癸钠作为首选药物预防癌症患者术后VTE的证据。临床通常用于LMWH过敏、HIT或LMWH无法获取时。

##### 5.3.3.3 维生素K拮抗剂

VKA（例如华法林）是癌症患者长期治疗VTE的一种选择^[6]^。但VKA存在以下显著缺陷：（1）治疗窗窄，个体变异大，需常规INR监测（international normalized ratio, INR），通过剂量调整维持靶INR 2.0-3.0；INR > 3.0可增加大出血风险。（2）多种药物和饮食因素与华法林相互作用，增加或降低华法林抗凝作用。因此，不推荐VKA用于胸部恶性肿瘤患者围术期VTE预防。（3）术前长期口服华法林的患者，其围术期大出血风险增大，通常术前停药，改用LMWH注射进行桥接抗凝。

##### 5.3.3.4 直接口服抗凝药物（NOACs）

鉴于目前关于NOACs用于胸部恶性肿瘤的安全性和有效性的数据很少，本共识不推荐使用NOACs进行胸部恶性肿瘤围术期VTE预防。

#### 5.3.4 药物预防禁忌证及注意事项

血栓预防禁忌证可分为绝对禁忌证和相对禁忌证两类：（1）绝对禁忌证：①近期活动性出血和凝血障碍；②骨筋膜间室隔综合征；③严重颅脑外伤或急性脊髓损伤；④血小板低于20×10^9^/L；⑤肝素诱导的血小板减少症（heparin induced thrombocytopenia, HIT）禁用肝素和低分子肝素；⑥孕妇禁用华法林。（2）相对禁忌证：①既往颅内出血；②有消化道出血史；③急性颅内损害或肿物；④急性出血史；⑤血小板计数降至（20-100）×10^9^/L；⑥类风湿或视网膜病变。（3）注意事项：①由于各肝素类抗凝药物（包括肝素及低分子肝素）的分子量、单位、剂量及活性不同，在预防过程中只能使用一种药物。不建议与另一种抗凝药物交替使用。每种药物都应遵循预防措施和不良反应的提示。②对于有肾或肝损害的患者，应谨慎调整剂量。严重肾损害患者不应使用低分子肝素和磺达肝癸钠。③出血是最严重的药物并发症，围术期应评估大出血的危险因素（表 7）^[18]^。

**7:** 大出血高危因素 Risk factors for bleeding complications

General risk factors
Active bleeding
Previous major bleeding
Known, untreated bleeding disorder
Severe renal or hepatic failure
Thrombocytopenia
Acute stroke
Uncontrolled systemic hypertension
Lumbar puncture, epidural, or spinal anesthesia within previous 4 h or next 12 h
Concomitant use of anticoagulants, antiplatelet therapy, or thrombolytic drugs
Procedure-specific risk factors
Abdominal surgery (Male sex, preoperative hemoglobin level < 13 g/dL, malignancy, and complex surgery defined as two or more procedures, difficult dissection, or more than one anastamosis)
Use of aspirin
Use of clopidogrel within 3 d before surgery
Thoracic surgery (Pneumonectomy or extended resection)
Having any of the above risk factors is a high risk of major bleeding.

**推荐意见7：对高危患者，围术期尽早开始进行VTE预防，包括药物预防和机械预防。**

**推荐意见8：药物预防推荐应用低分子肝素，预防时长高危患者7 d-10 d，极高危患者30 d。**

### 5.4 下腔静脉滤器（inferior vena cava filter, IVC）

不建议癌症患者常规植入IVC作为预防措施，即使是PE高危患者也不推荐常规使用。适应证：近端DVT，全剂量抗凝治疗禁忌证或者近期接受大手术的患者，仅推荐短期应用，不建议长期放置^[6, 25]^。

**3:**
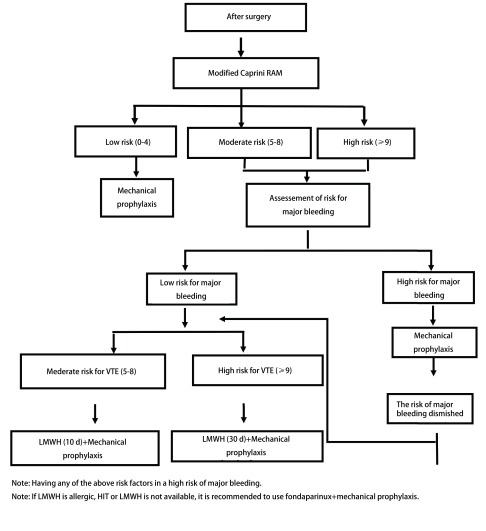
术后VTE风险再评估流程图 Algnothm of post-operative VTE re-assessement

## 6 小结

胸部恶性肿瘤患者围术期VTE的发生率较高，但其发病隐匿，常无症状或症状不典型，易被忽视，临床应予以高度重视。VTE的早期识别、早期诊断和规范预防可以有效降低VTE的风险，因此应对所有胸部恶性肿瘤的住院患者进行VTE风险评估。对于所有患者应根据其风险分层，进行规范的围术期VTE预防。

**中国胸外科静脉血栓栓塞症研究协作组**

**组长**

李辉（首都医科大学附属北京朝阳医院胸外科）

**执笔专家**

李辉（首都医科大学附属北京朝阳医院胸外科）

姜格宁（同济大学附属上海肺科医院胸外科）

**审定专家组（按姓氏拼音字母顺序）**

陈椿（福建医科大学附属协和医院胸外科），陈克能（北京大学肿瘤医院胸外一科），陈海泉（复旦大学附属肿瘤医院胸外科），陈军（天津医科大学总医院肺部肿瘤外科），崔向丽（首都医科大学附属北京朝阳医院药事部），车国卫（四川大学华西医院胸外科），付向宁（华中科技大学附属同济医院胸外科），高树庚（中国医学科学院肿瘤医院胸外科），何建行（广州医科大学附属第一医院胸外科），胡坚（浙江医科大学附属第一医院胸外科），姜格宁（同济大学附属上海肺科医院胸外科），李辉（首都医科大学附属北京朝阳医院胸外科），李拥军（北京医院血管外科），李玉萍（同济大学附属上海肺科医院重症监护室），李小飞（空军军医大学唐都医院胸外科），梁朝阳（中日友好医院胸外科），苗劲柏（首都医科大学附属北京朝阳医院胸外科），沈蕾（同济大学附属上海肺科医院外科重症监护室），孙伟（新疆医科大学附属肿瘤医院胸外科），佟宏峰（北京医院胸外科），王群（复旦大学附属中山医院胸外科），许顺（中国医科大学附属第一医院胸外科），薛涛（东南大学附属中大医院胸外科），于振涛（天津医科大学附属肿瘤医院胸外科），杨梅（四川大学华西医院胸外科），杨媛华（首都医科大学附属北京朝阳医院呼吸及危重症医学科），张临友（哈尔滨医科大学附属第二医院胸外科），张兰军（中山大学附属肿瘤医院胸外科），张逊（天津市胸科医院胸外科），赵珩（上海交通大学附属胸科医院胸外科），支修益（首都医科大学附属宣武医院胸外科）

**编写秘书**

苗劲柏（首都医科大学附属北京朝阳医院胸外科）
